# Attitudes and Beliefs of Portuguese and American Nursing Students about Patients’ Sexuality

**DOI:** 10.3390/healthcare10040615

**Published:** 2022-03-25

**Authors:** Margarida Sim-Sim, Vicki Aaberg, Hélia Dias, Ermelinda Caldeira, Cinzia Gradellini, Daniela Mecugni, Sagrario Gomez-Cantarino

**Affiliations:** 1 Nursing Department, University of Evora, Comprehensive Health Research, Integrated Researcher (CHRC), Escola Superior de Enfermagem S. João de Deus, 7000-811 Evora, Portugal; msimsim@uevora.pt (M.S.-S.); ecaldeira@uevora.pt (E.C.); 2 School of Health Sciences, Seattle Pacific University, Seattle, WA 98119, USA; aaberv@spu.edu; 3 Instituto Politecnico de Santarém, Escola Superior de Saúde, 2005-075 Santarém, Portugal; helia.dias@essaude.ipsantarem.pt; 4 EdSex Project, University of Modena and Reggio Emilia, Azienda Unità Sanitaria Locale-IRCCS of Reggio Emilia, 42122 Reggio Emilia, Italy; daniela.mecugni@unimore.it; 5 Faculty of Physiotherapy and Nursing, Toledo Campus, University of Castilla-La Mancha, Avda Carlos III, s/n, 45071 Toledo, Spain; sagrario.gomez@uclm.es; 6 Health Sciences Research Unit: Nursing (UICISA: E), The Nursing School of Coimbra (ESEnfC), 3004-011 Coimbra, Portugal

**Keywords:** nursing, health personnel, clinical competence, sexuality, intervention

## Abstract

Nursing school graduates must be prepared to interact comfortably and effectively with patients about their sexual health. This study analyses the attitudes and beliefs about patient sexuality held by Portuguese and American nursing students. Objective: In Portuguese and American nursing students, (1) we analyzed students’ attitudes and beliefs towards sexuality using the Sexuality Attitudes and Beliefs Survey (SABS); (2) we identified nationality, socio-demographic information, and affective-sexual beliefs and attitudes. Method: Quantitative, cross-sectional study; convenience sample of 296 students (63.2% Portuguese; 36.8% American); mean age: 21.9 years (SD = 3.12); two-way ANOVA and multiple correspondence analyses were performed. Results: Attitudes and beliefs toward sexuality: Portuguese women are more liberal than men, contrary to American students. Among both nationalities, participants with multiple sexual partners held more conservative attitudes. Sexual orientation: bisexual American students and homosexual Portuguese students are conservative. Multiple correspondence analysis revealed two profiles: (1) Portuguese students: liberal-tolerant in attitudes towards patient sexuality, live with family/roommate, 18 to 21 years old, no or one sexual partner; (2) US students: traditionalist attitudes towards patient sexuality, share house, 22 and 23 years old, multiple partners. Conclusion: Human sexuality must be addressed in nursing education curricula.

## 1. Introduction

Nursing students, while studying to work in their profession, perform care interactions in a real context. They deal with the patient’s body and intimate issues and must engage with expressions of people’s sexuality. 

At the beginning of their undergraduate education, students’ knowledge about sexuality is rooted in primary sources such as family, friends and the media [1,2]. At this stage, attitudes and beliefs tend to express traditionalism [3,4]. The social and family environment from which they come plays a determining role by offering values and life perspectives. Students from conservative families or those belonging to religious denominations are more traditionalist [2,5]. 

Sexual health is an important area for holistic care [3] and requires intentional training in nursing curricula [3,6]. The undergraduate curricula of European and American nursing education programs cover similar subjects, including sexual orientation, gender and identity [6]. However, as the inclusion of sexuality issues is controversial in some settings, the need for sexuality content in nursing education is clear and demands consistent use of pedagogical curricular strategies for effective inclusion in all settings [6,7,8,9,10].

Nursing students consider addressing sexuality a requirement of the profession [1,11,12]. In addition, teachers emphasize the need to educate students on positive attitudes in order to be more effective in dealing with patients [10,13]. Some students criticize the curricula, stating that there is little theoretical preparation and little training for the clinical experiences [3,9], especially with regard to lesbian, gay, bisexual, and transgender (LGBTQ) issues [1,14]. In clinical practice, they report a lack of time to address patients’ sexual concerns and find that patients do not expect nurses or students to address sexual issues [7,15]. Studies reveal that students feel anxiety and worry about how they may be viewed by their colleagues when deciding to address patients regarding sexuality [12]. The students’ resistance or difficulty worsens when the patient has a non-heterosexual orientation [16]. Although they may hold favourable attitudes, they feel discomfort and are reluctant to initiate the subject [1,5,11,16]. 

In Portugal, undergraduate nursing students’ attitudes are less permissive and more conservative compared to students studying other health professions [17]. On the other hand, American studies identify more favourable attitudes and better knowledge and self-efficacy after specific training of undergraduate students in sexual health [18]. When the same instrument was applied to younger (accelerated program) American students, they held more conservative attitudes and beliefs than older (traditional program) undergraduate students in addressing the sexual concerns of patients [15]. In nursing students, research has used several instruments [17,19] for analysing attitudes, opinions and beliefs towards patient sexuality. However, it is rare that the same study takes place in samples of different countries and in diverse languages. In fact, the application of a single instrument, validated for different cultures, guarantees a reliable analysis of the construct. The objectives are (1) to analyse the attitudes and beliefs towards sexuality of the patient through the application of the Sexuality Attitudes and Beliefs Survey (SABS) and (2) to identify the profile of the students, considering characteristics such as nationality and socio-demographic and affective-sexual data.

## 2. Materials and Methods

This is a quantitative study of inferential character and uses a transversal approach with a convenience sample, with the participation of 296 undergraduate nursing students. Portuguese students attended state schools and American students attended private schools. Data from the Portuguese students were collected online, through the Lime-survey platform, by sending 340 emails to students from Évora and 375 emails to students from Santarém. The data from the American students, located in Seattle, were collected on paper from 135 students. 

### 2.1. Data Collection Instrument

The instrument applied consisted of two sections: (a) sociodemographic data and (b) SABS scale in the original version [19] or in the version validated for Portuguese [20]. The scale is unidimensional, and the original form consists of a set of 12 items with a Cronbach’s alpha coefficient of 0.75 and retest of 0.82 [19]. In the Portuguese version, the observation of the psychometric properties implied the removal of item 3, thus resulting in 11 items. In this version, the Cronbach’s alpha coefficient was 0.72 and the test-retest was 0.80 [20]. In the current study, the subsample of Portuguese students (11 items in the SABS scale) shows a Cronbach’s alpha value of 0.67, and the subsample of American students (12 items in the SABS scale) demonstrates a Cronbach’s alpha value of 0.66.

The items are presented on a six-position scale, ranging between 1 (i.e., strongly disagree) and 6 (i.e., strongly agree). Items 1, 2, 4, 6, 8, 10 and 12 are reverse-formulated and are scored accordingly. Higher scores indicate stronger and more limiting attitudes and beliefs about addressing sexuality with the patient. In the current study, the response score on the SABS was analysed using the mean. 

### 2.2. Ethical Procedures

The invitation to participate was presented face to face in the classroom, at a date prior to data collection. In the data collection instrument, the first page of both the online and paper versions presented the informed consent. In view of the student’s inability to manually sign the online version, the first question was mandatory to answer. He/she could not progress to the questionnaire without explicitly ticking (i.e., yes versus no) to confirm consent. In the paper version, consent was signed in person. The questionnaires were anonymised, with no elements that could link the respondent to the response.

The study was submitted to and approved by the Ethics Committee for Research in the Areas of Human Health and Welfare of the University of Évora (Registration No. 18175). 

### 2.3. Data Analysis

Statistical analysis was performed using the Statistical Package for the Social Sciences (IBM SPSS Statistics for Windows, Version 24^®^). The distribution of the sample, according to nationality showed normality (KS = 0.055; df = 187; *p* = 0.200; KS = 0.054; df = 109; *p* = 0.200) in the Portuguese and Americans, respectively. We opted for a parametric test approach, performing two-factor ANOVA analysis of variance and multiple correspondence analysis. Values are presented in mean and standard deviation, and the significance level is *p* < 0.05.

## 3. Results

A total of 296 students participated, of whom 187 (63.2%) were Portuguese and 109 (36.8%) American. The response rate in Évora was 28.5% (i.e., 340 sent vs. 97 completed responses), in Santarém was 24% (i.e., 375 sent vs. 90 completed responses) and in Seattle was 80.7% (i.e., 135 invited vs. 109 completed).

The age of participants ranged from 18 to 40 years, with a mean of 21.9 years (SD = 3.12) and a mode of 22 years. The representation of males compared to females was lower in both schools (*n* = 25: 13.4% vs. *n* = 162: 86.6% in the Portuguese; *n* = 15: 13.8% vs. *n* = 94: 86.2% in the Americans). The majority were in the 22–23 age group (*n* = 115: 38.9%). Regarding current residence, most Portuguese lived with their parents (*n* = 70: 37.4%), while the Americans lived with their peers in a rented house (*n* = 38: 34.9%).

Regarding sexual orientation, most defined themselves as heterosexual (*n* = 274: 92.6%) in the total sample. The majority of the students stated that they currently had a sexual partner (*n* = 152: 51.4%), while 76 (25.7%) stated they were currently not in a relationship and 64 (21.6%) had never had an affective-sexual relationship. The sociodemographic, sexual orientation and affective-sexual relationship data are shown in Table 1.

**Figure 1:**
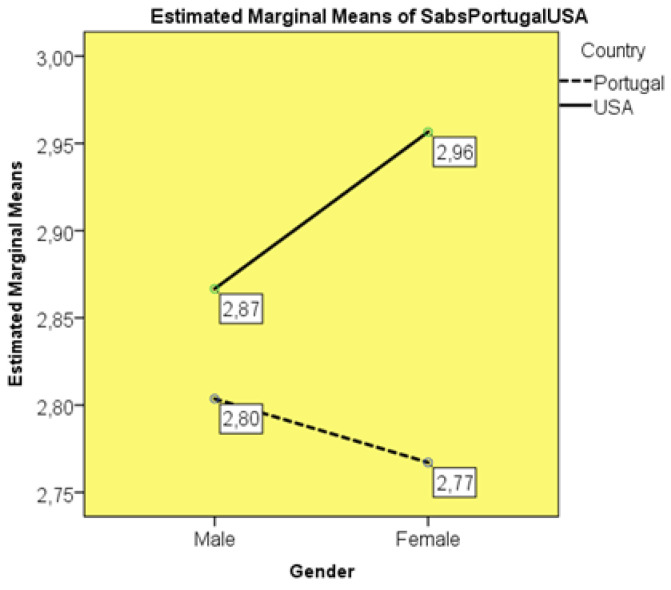
SABS considering students’ nationality and gender.

Through a two-factor ANOVA (gender and nationality), having verified the equality of variances by Levene’s test (*p* = 0.261), it was observed, without significant differences (F_(3:292)_ = 2.119; *p* = 0.098), that American students had higher mean scores on the SABS (M = 2.91; SD = 0.081) than Portuguese students (M = 2.78; SD = 0.063). Among Portuguese students, females show less conservative attitudes and beliefs towards the sexuality of the patient (M = 2.77; SD = 0.057) than males (M = 2.80; SD = 0.667). However, in American students, males are more liberal in their approach to patient sexuality than females (M = 2.86; SD = 0.410 vs. M = 2.95; SD = 0.608), according to Figure 1.

**Figure 2:**
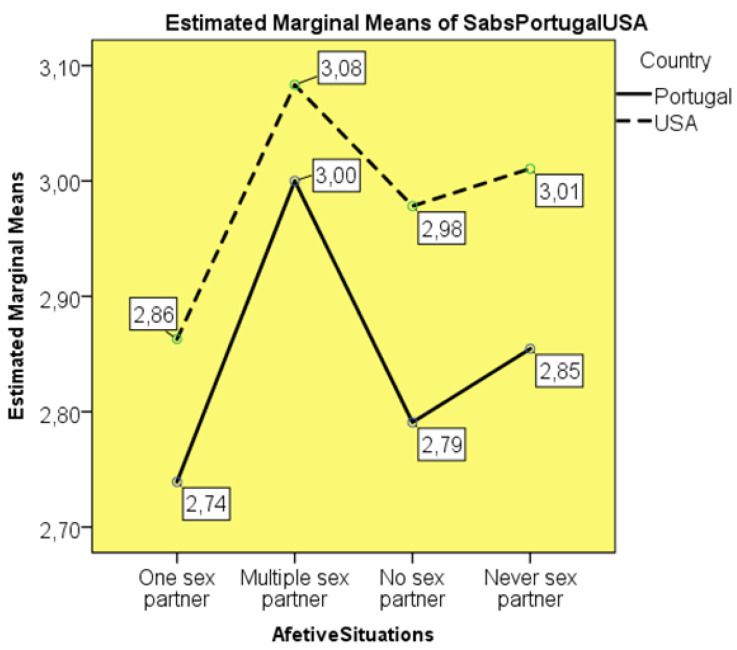
ABS considering students’ nationality and affective relationship.

A two-way ANOVA test was performed, using the SABS scale and the students’ nationality and the affective relationships they reported. The homogeneity of variances was verified in Levene’s test (*p* = 0.945). It was observed without significant differences (F_(7:288)_ = 1.239; *p* = 0.281), that American students held numerically higher averages in the SABS, that is, more limiting attitudes and beliefs in the approach to sexuality of the patient compared to the Portuguese students (M = 2.98; SD = 0.113 vs. M = 2.84; SD = 0.111). On the other hand, in both nationalities, the most conservative participants in their approach to patient sexuality were those with multiple sexual partners (M = 3.08; SD = 0.415 American; M = 3.00, SD = 0.415 Portuguese). The more liberal participants revealed having only one sexual partner (M = 2.86, SD = 0.087 and M = 2.74, SD = 0.057) (Figure 2).

**Figure 3:**
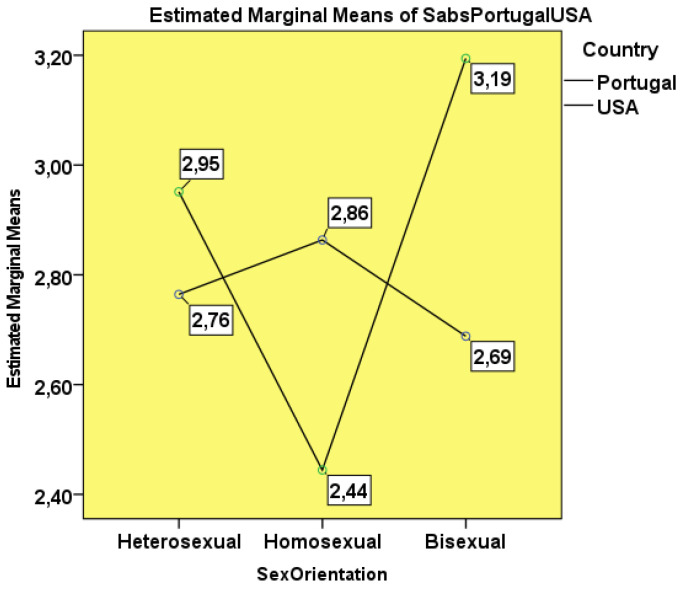
SABS considering students’ nationality and sexual orientation.

Considering the students’ sexual orientation (heterosexual, homosexual and bisexual), excluding the three participants who did not define themselves, a new two-factor ANOVA test (i.e., nationality and sexual orientation) was performed. Levene’s test showed equality of variances (*p* = 0.086). Without significant differences (F_(5:287)_ = 1.906; *p* = 0.093), American students showed numerically higher means in the attitudes and beliefs towards sexuality of the patient when compared to the Portuguese (M = 2.863, SD = 0.160 vs. M = 2.772, SD = 0.109). It was observed that the most conservative about patient sexuality were the American students with a bisexual orientation (M = 3.19, SD = 0.337) and the least traditional were those who declared themselves homosexual (M = 2.44, SD = 0.337). In contrast, in the Portuguese, the most conservative were the students with a homosexual orientation (M = 2.86, SD = 0.238) (Figure 3).

One focus of this study was to identify the profile of nursing students related to attitudes toward patient sexuality, considering underlying characteristics like nationality, sociodemographic data (age, gender, type of residence where they live) and affective relationships. A multiple correspondence analysis (MCA) was developed to process the responses. 

Before starting the analysis, the maximum and minimum scores on the SABS scale (Min = 1.36 and Max = 4.64) were considered, and three groups with the same range were formed, with cut-off points at 2.46 and 3.55. The SABS was thus defined as the liberal group (scale score between 1.36 and 2.45), the tolerant group (scale score between 2.46 and 3.54) and the traditionalist group (scale score between 3.55 and 4.64).

In the first exploration, the maximum number of dimensions (i.e., nine dimensions) was used to observe the behaviour of the inertia values (i.e., eigenvalues), according to Table 2. The first two dimensions have supremacy, with higher eigenvalues (i.e., 1.907, 1.371). A two-dimensional solution was then used. The total inertia became 0.546. The variance explained for Dimension 1 is 31.78% and for Dimension 2 is 22.85%.

### Discrimination Measures

The discrimination measures of the variables residence, affective relationship and nationality have higher values in Dimension 1 (i.e., respectively, 0.571, 0.337 and 0.540).

**Figure 4:**
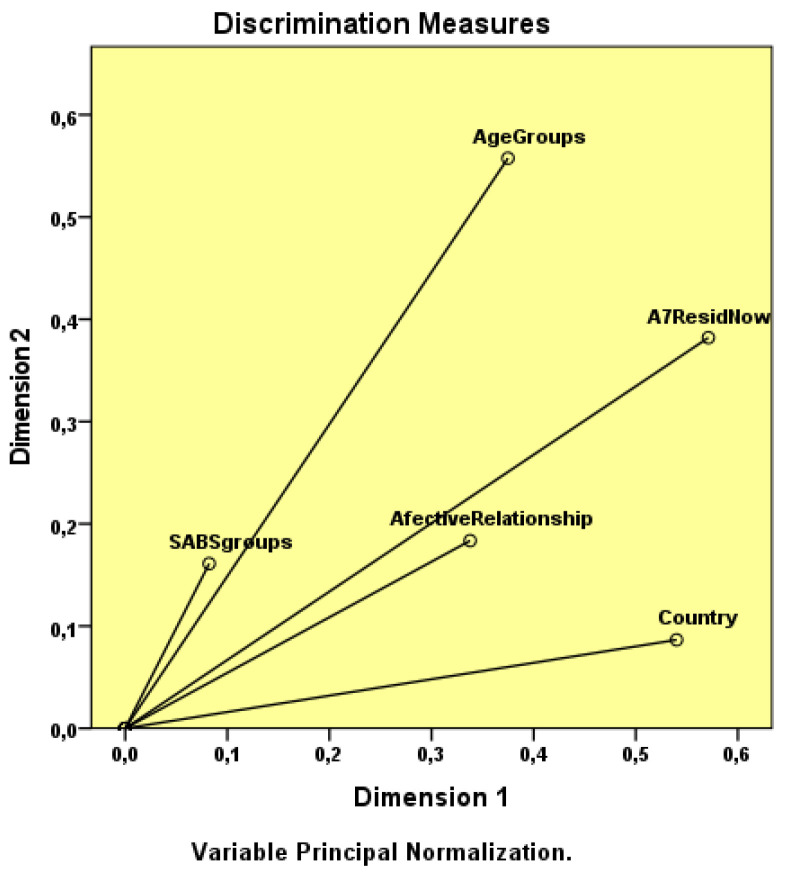
Discriminant measures.

In Dimension 2, the discrimination measures of the variables SAB groups and age groups have higher values (i.e., SABS groups = 0.159; Age Groups = 0.563). The gender indicator is not relevant in any of the dimensions, as revealed in Table 3, and in the observation of the discrimination measures chart, since the sex variable is very close to the origin (Figure 4).

**Figure 5:**
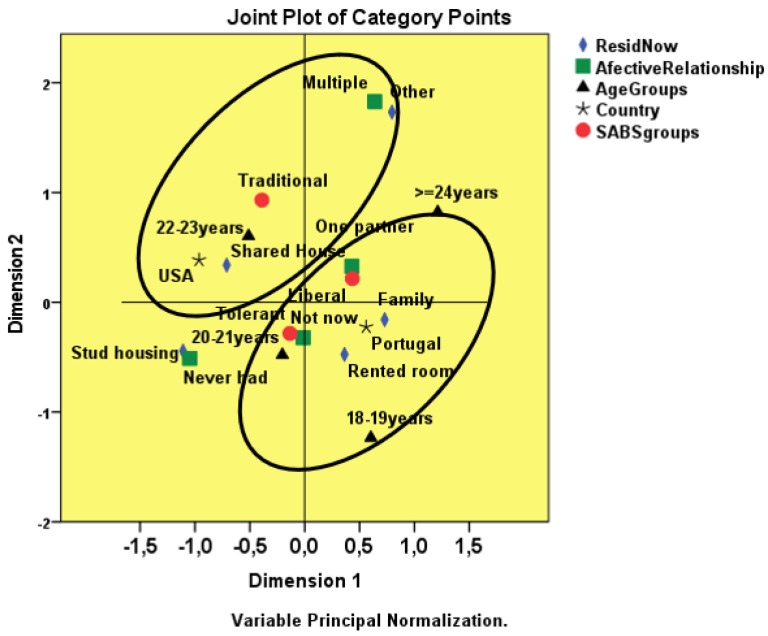
Joint plot of category points resulting from the multiple analysis.

If we remove the gender variable from the analysis, the distribution and grouping of the categories suggests that the American students have a traditionalist profile manifested by students aged 22–23 years, living in rented accommodation with colleagues and with multiple affective relationships. In the Portuguese students, the profile oscillates between liberal and tolerant attitudes toward engaging with the patients’ sexuality, falling into lower age groups, living in a family house or rented room, with no current affective-sexual relationship or with an exclusive partner (Figure 5).

## 4. Discussion

The objective of this study was to examine Portuguese and American nursing students’ attitudes toward patients’ sexuality and define their profile. The different proportion of answers obtained in the Portuguese versus American institutions reveals the influence that the collection method may have on the final data set. The response rates in the online application coincide with previous studies, which refer to a representation between 20% and 47%, while in the collection via paper, the sample is higher than those found in the literature, which vary between 32.6% and 75% [21]. Although the use of the internet increases the dissemination to and access of potential respondents, as well as the suitability for personal schedules and privacy, compared to the classroom during school hours, the response rates do not seem to reflect these advantages. The overload of requests for collaboration via the internet may be a demotivating factor. Perhaps the direct and personal stimulus of the researcher, providing a paper questionnaire and requesting collaboration from the students, is the cause of a more successful response rate. On the other hand, the online data collection route should be cultivated, as it favours the preservation of the environment and the dematerialisation of communication, and it saves financial resources. In fact, in addition to the non-countable expenses to the environment, the paper-based application would be a considerable burden on Portuguese students.

Gender representation is similar in both sets of participants, favouring women. This was expected, as nursing is a career widely chosen by women. In this profession, the nature of caring imprints social attributes associated with femininity. In addition, there may be higher social expectation for women to be nurses, thereby feminising the profession [22]. The lower number of male students may be due to ambivalence between the call to the profession and the construct of professional identity. Some studies show that there are barriers to student development, including the lack of a clear and acceptable public image, which suggests the idea that the option for nursing is not appropriate for men [23]. Gender stereotypes in professions may induce biases, since performance expectations are misrepresented. Not only in the population but also among nursing students, there are gender stereotypes [22] that can negatively affect students’ professional paths. Professions that, like nursing, are associated overwhelmingly with one gender present a risk that some members of the profession may be devalued when the traditional gender boundaries are broken [24]. 

Although heterosexuality is the orientation most frequently mentioned by the participants, the fact that some students reported other orientations reveals that they are in the process of recognizing and making known the way they feel and live their sexuality. The entry into university approximately coincides with the leaving of the parents’ home, a phase of searching for adult identity in which young people tend to free themselves from family norms. In this context, a positive view of orientations other than heterosexuality grows and becomes more acceptable [25] and feelings of self-marginalisation can be reduced.

The affective-sexual relationships recognised by the participants, in the sense of romantic relationships, follow the juvenile framework, when the adolescent crisis is overcome [26]. The type of involvements and the expressions of gender identity in the current study correspond to those identified in the literature [26,27]. In fact, during university education there are typical behaviours (i.e., exclusivity, short relationships, sequential monogamies, multiple partners), which may reflect development, opportunities or experimentation.

### 4.1. Gender vs. Nationality in the SABS

The SABS results in the two-way ANOVA between the factors of sex and nationality point to a difference in female students, with American students demonstrating the most conservative results, and Portuguese female students demonstrating the most liberal. The interpretation requires care to avoid xenophobic allocations or approximations to nationalism, machismo or marianism. The similar SABS scores for Portuguese and American men can be interpreted from a gender perspective, as men may be less subject to social influences than women. In fact, the role norms with which men and women from different cultural, ethnic and religious traditions invest in care are different [28]. The results seem to be rooted in the pathway of nursing education. The admission of more male students, older people, individuals with different marital status and families, and emigrants or their descendants has broken the conventional female profile [28]. Moreover, although the profession retains a female majority, the sexual division of care is blurring [29]. The higher score of American women in the SABS score suggests that private university institutions may favour a more conservative education. In the Portuguese polytechnic environment, there may be greater openness in this area of patient care. Controversy, however, may still reside in the eventual perception of the students regarding the client’s responses, since in some cultures, men prefer male caregivers, while women prefer female [28,29]. A bias related to social desirability may still be present, as American and Portuguese students responded to what is expected of the good caregiver [29].

### 4.2. Affective-Sexual Relationship vs. Nationality

The SABS results in the two-way ANOVA between the factors of type of affective-sexual relationship and nationality suggest the dominance of a hidden curriculum, prior and simultaneous to the nursing training process, to which both samples are exposed. There may be social stereotypes in which both the experience of one’s own sexuality and opinions about the sexuality of others are rooted. On the other hand, this suggests that in spite of the sociocultural surroundings, there is an individual and singular path in the development of the student. Not having experienced affective-sexual relationships and not having a partner or being in an exclusive relationship seem to transmit a greater openness to the sexuality of the others and to consider the patient as a total being. The participants with multiple partners, on the other hand, by showing more barriers, suggest less maturity, and less affective-emotional development. Perhaps these individuals are still trapped in a late adolescence, which prolongs experimentation [26]. 

### 4.3. Sexual Orientation vs. Nationality

Students coming from conservative, religious backgrounds are more traditionalist [2,5] in their attitudes toward sexuality. The relationship between conservative religious beliefs and attitudes and beliefs toward patient sexuality need to be explored in further detail. These authors describe the results obtained in the ANOVA with the two factors of sexual orientation and nationality.

### 4.4. The SABS Profile in Portuguese and American Students 

The MCA revealed relations of interdependence, and thus profiling, between the student attitudes to addressing sexuality in patient care and a series of sociodemographic variables, including affective relationship, nationality, place of residence and age. The MCA, although with exploratory purposes, was useful in this analysis, as it involved multifaceted structures rooted in the cultural environment [30]. It allowed us to observe the association between the categories of different variables, thus defining profiles. This is useful for understanding the vision of the participants and for emphasizing to teachers and clinical supervisors the students’ needs for guidance in the care of patients as unique and total beings who live their sexuality independently of their health or illness status. It is important to emphasize the patient’s sexuality as a human dimension in the teaching–learning process of nursing students, both in theoretical and clinical approaches [15]. The identified profiles highlight aspects that call for teaching strategies so that students can develop. These profiles are consistent with studies on university students from the American continent, where uninhibitedness (i.e., multiple partners) is evident when it comes to personal sexuality [31] but, contradictorily, the same individuals report more conservative sexual attitudes with regard to patient care, suggesting that their attitude is rooted in their sociocultural background. 

With regard to the Portuguese students, the profile suggests a liberal attitude. However, they are younger students and thus more susceptible to prescriptive teaching models with a biomedical root. In Portugal, although under the European guidelines, it is not clear in the undergraduate nursing study plans which subjects include the patient’s sexuality as an explicit and concrete teaching subject. The approach to the sexuality of the patient mainly involves strategies based on the transmission of anatomical and physiological knowledge. In fact, the profile of the Portuguese students participating in this study is in agreement with semantic representations of sexuality, which are oriented toward the normative in the psychophysiological dimensions [32,33,34]. 

We may suspect that in both profiles the predisposition to approach the sexuality of the patient reverts into postponement or is even contradictory. Although the students identify their roles, they hesitate to follow through [35,36]. Considering sexuality as an area of care, but having reservations and subsequently not carrying out the care, underlines beliefs or barriers.

## 5. Conclusions

Despite the current eroticized society, nursing students’ attitudes towards patient sexuality seem to be anchored in traditionalism. On the other hand, a certain cleavage seems to emerge between the recognition of the patient’s sexuality as an area to be considered in care provision and the predisposition to address it in clinical practice. In fact, the profile of both groups of students reveals openness to providing this care yet hesitation to do so. 

Although the students identify their role, they hesitate to play it. It will be important to think of undergraduate training programmes in nursing as the beginning of the construction of competencies, so that the sexuality of the patient is assumed as a dimension of human life. Such programmes may need evaluation indicators oriented toward the theme of sexuality and strategies for the incorporation of this content. These strategies may include small group work with discussion of cases or dilemmas, interview training for data collection, simulation, autoscopies, and role playing under the guidance of a supervisor.

Considering sexuality as an area of care, but having reservations and not following through on the care, reveals underlying beliefs or barriers that, in the profession, are contradictory, since nursing mandates holistic care. The inclusion of skills to learn to care for the patient in this area seems urgent. The current results show the need for formative programs where the student’s individual, social and cultural dimensions should be examined for the influence they may have on students’ ability to engage in the topic of sexuality with patients. 

## 6. Limitations

The non-randomised sample prevents generalisation of the results. Data collection through two formats (online and face to face) may imprint bias. Another weakness of the current study is the heterogeneity of the sample, with an age range of 18–40 years and in various years of training.

## Figures and Tables

**Table 1:** Characteristics of the participants.

Category	Characteristic	Portuguese	American	Total
	*n* (%)	*n* (%)	*n* (%)	
Sex	Male	40 (13.5)	25 (13.4)	15 (13.8)
	Female	256 (86.5)	162 (86.6)	94 (86.2)
Age	18–19 years old	51 (17.2)	45 (24.1)	6 (5.5)
	20–21 years old	92 (31.1)	57 (30.5)	35 (32.1)
	22–23 years old	115 (38.9)	51 (27.3)	64 (58.7)
	23–40 years old	38 (12.8)	34 (18.2)	4 (3.7)
Living at	Family home	85 (28.7)	70 (37.4)	15 (13.8)
	Academic residency	53 (17.9)	22 (11.8)	31 (28.4)
	Rented room	65 (22.0)	47 (25.1)	18 (16.5)
	Rented house with colleagues	67 (2.6)	29 (15.5)	38 (34.9)
	Other	26 (8.8)	19 (10.2)	7 (6.4)
Sexual Orientation	Heterosexual	274 (92.6)	171 (91.4)	103 (94.5)
	Homosexual	9 (3.0)	6 (3.2)	3 (2.8)
	Bisexual	10 (3.4)	7 (3.7)	3 (2.8)
	Not defined	3 (1.0)	3 (1.6)	-
Sexual partner	Never had sex partner	64 (21.6)	25 (13.4)	39 (35.8)
	No sex partner now	76 (25.7)	53 (28.3)	23 (21.1)
	One sex partner now	152 (51.4)	107 (57.2)	45 (41.3)
	Multiple sex partners	4 (1.4)	2 (1.1)	2 (1.8)
Total		296	187	109

**Table 2:** Two-dimensional solution.

Model Summary				
Variance Accounted for				
Dimension	Cronbach’s Alpha	Total (Eigenvalue)	Inertia	% of Variance
1	0.571	1907	0.318	31.778
2	0.325	1371	0.229	22.854
Total		3278	0.546	
Mean	0.468 ^a^	1639	0.273	27.316

^a^ Mean Cronbach’s Alpha is based on the mean Eigenvalue.

**Table 3:** Discrimination measures of variables entered in the 2-dimensional model.

Discrimination Measures			
	Dimension		Mean
	1	2	
ResidNow	0.571	0.376	0.474
AfectiveRelationship	0.337	0.186	0.262
Country	0.540	0.087	0.314
AgeGroups	0.375	0.563	0.469
SABSgroups	0.082	0.159	0.121
Gender	0.000	0.001	0.000
Active Total	1.907	1.371	1.639
% of Variance	31.778	22.854	27.316

## Data Availability

Not applicable.
